# Disappearance, division, and route change of excitable reaction-diffusion waves in deformable membranes

**DOI:** 10.1038/s41598-023-33376-9

**Published:** 2023-04-17

**Authors:** Hiroshi Noguchi

**Affiliations:** Institute for Solid State Physics, University of Tokyo, Kashiwa, Chiba 277-8581 Japan

**Keywords:** Membrane biophysics, Biological physics, Nonlinear phenomena

## Abstract

Shapes of biomembrane in living cells are regulated by curvature-inducing proteins. However, the effects of membrane deformation on signal transductions such as chemical waves have not been researched adequately. Here, we report that membrane deformation can alter the propagation of excitable reaction-diffusion waves using state-of-the-art simulations. Reaction waves can induce large shape transformations, such as membrane budding and necking, that erase or divide the wave, depending on the curvature generated by the waves, feedback to the wave propagation, and the ratio of the reaction and deformation times. In genus-2 vesicles, wave division occurs at branching points and collided waves disappear together. We demonstrate that the occasional disappearance of the waves can alter the pathway of wave propagation. Our findings suggest that membrane deformation and reaction waves can together regulate signal transductions on biomembranes.

## Introduction

Animal tissues and cells are subjected to chemical reaction waves that are often involved in signal transductions^1–4^. The chemical waves were observed in the locomotion of cells and were reproduced by reaction-diffusion (RD) equations^5^. The patterns on the skins of mammals, reptiles, and fishes can be understood by RD as Turing patterns^6,7^. Recently, RD dynamics on curved surfaces has gained considerable attention, since the surfaces of tissues and biomembranes are not flat^8,9^. Reflection asymmetry induces pattern propagation in axisymmetric surfaces, even in the condition that Turing patterns are stable at flat surfaces^10^. A growing surface with a fixed shape modifies the patterns^8,11^. However, the curvature effects remain largely unexplored.

Many types of proteins regulate the shape of biomembrane in living cells. In particular, the binding of curvature-inducing proteins, such as clathrin and Bin/Amphiphysin/Rvs (BAR) superfamily proteins, induces a local membrane curvature^12–16^. The binding and unbinding of many of these proteins are activated or inhibited by adenosine triphosphate (ATP) and guanosine triphosphate (GTP) hydrolysis. Recently, experiments have been conducted to observe the membrane deformations accompanied by waves of F-BAR and Min proteins^17–19^. The interaction with actin filaments is also involved in the membrane deformation waves^2,3,17,20^. However, the effects of surface deformation on RD have been little elucidated. Despite the importance of mechanochemical coupling, the small deformation of membrane has only been considered theoretically^2^.

Previously, we have studied RD dynamics accompanied by large membrane deformation using dynamically triangulated membrane simulations^21–23^. The study revealed that membrane deformation stabilizes Turing patterns through the formation of budded and multi-spindle-shaped vesicles^21^. Moreover, we have successfully reproduced the self-oscillation of vesicle shapes as seen in the reconstructed Min system in liposomes^22^ and revealed that the deformation of tubular membranes can stop wave propagation and generate azimuthally undulated waves^23^. However, excitable waves exhibit only a small membrane deformation owing to relatively fast wave propagation in Ref. ^23^. Herein, we have extended our study to examine the coupling of an excitable wave and deformation of tubular membranes (straight and branched tubes) in various conditions. Tubular membrane structures are observed in plasma membranes (e.g., axons of neurons) and also intracellular organelles (e.g., endoplasmic reticulum (ER) and mitochondrion)^12,14^. We observed various behaviors of the waves caused by the membrane deformation. A reentrant transition occurs from wave propagation to disappearance with an increasing time ratio of wave propagation and membrane deformation. Wave division and rotation appear in addition to wave disappearance. The propagation pathways can be altered by membrane deformation in a tubular network.

**Figure 1:**
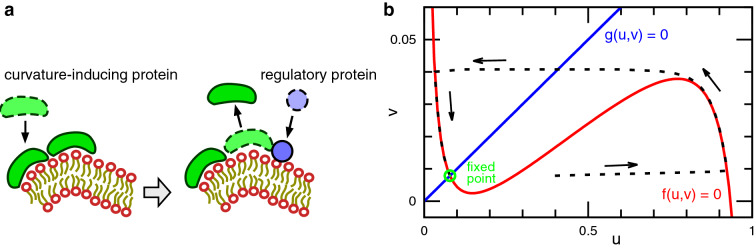
Reaction model used in this study. (**a**) Schematic of the binding/unbinding of two curvature-inducing proteins and one regulatory protein to a membrane. (**b**) Nullclines of the reactions (Eqs. (2) and (3)) for the concentration of curvature-inducing proteins *u* and regulatory proteins *v*. The black dashed line represents a trajectory of excitation. The green circle represents the fixed point.

**Figure 2:**
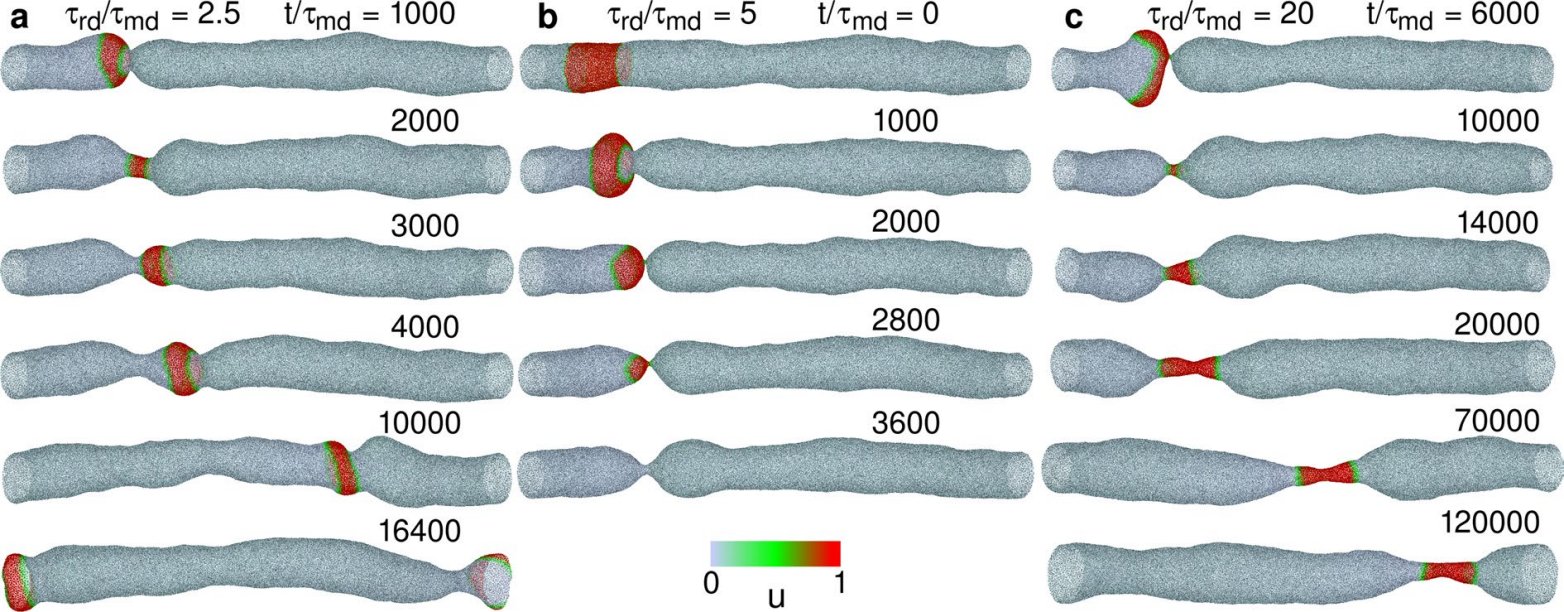
Sequential snapshots of RD waves in tubular membranes at $G=0.002$, $\kappa _1/\kappa _0 = 4$, and $C_0R_{\textrm{tube}}=3$. (**a**) Wave propagates for $\tau _{\textrm{rd}}/\tau _{\textrm{md}}=2.5$. (**b**) Wave disappears at $t/\tau _{\textrm{md}}=3500$ for $\tau _{\textrm{rd}}/\tau _{\textrm{md}}=5$. (**c**) Wave propagates, thus forming a narrow tube of the bound membrane for $\tau _{\textrm{rd}}/\tau _{\textrm{md}}=20$. The concentration *u* of curvature-inducing proteins is indicated in different colors (see the color bar in the bottom center). The initial state (top panel) in (**b**) is also used for the membrane dynamics shown in (**a**) and (**c**).

**Figure 3:**
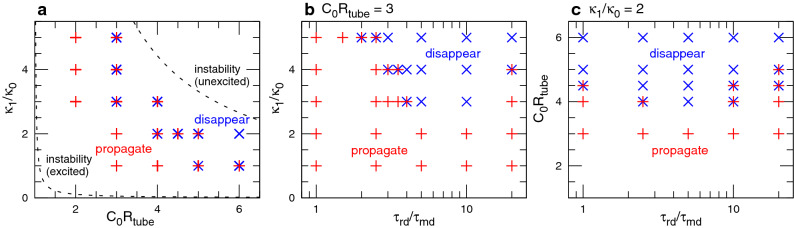
Phase diagrams at $G=0.002$. The symbols $+$ and $\times$ indicate the wave propagation and disappearance, respectively. (**a**) $C_0R_{\textrm{tube}}$ vs. $\kappa _1/\kappa _0$. The data for $\tau _{\textrm{rd}}/\tau _{\textrm{md}}=1$, 2.5, 5, and 20 are overlayed at each point. The upper and lower dashed lines represent the upper thresholds, indicating that the cylindrical membrane shape is stable for the unexcited ($u=0.07$) and excited ($u=0.9$) membranes, respectively. (**b**) $C_0R_{\textrm{tube}}=3$. (**c**) $\kappa _1/\kappa _0=2$. Both the symbols are overlayed when both dynamics are observed in six simulation runs.

**Figure 4:**
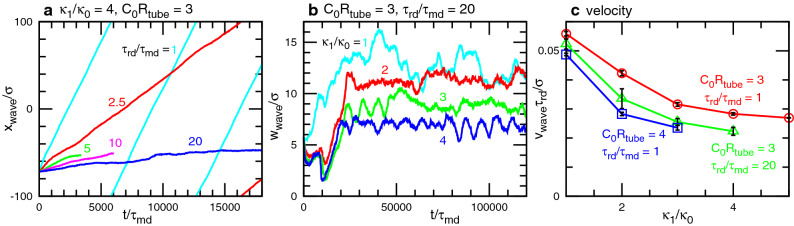
Time evolution of RD waves at $G=0.002$. (**a**) Center position $x_{\textrm{wave}}$ of the wave along the membrane (*x*) axis at $\kappa _1/\kappa _0 = 4$ and $C_0R_{\textrm{tube}}=3$. (**b**) Wave width $w_{\textrm{wave}}$ along the membrane axis at $C_0R_{\textrm{tube}}=3$ and $\tau _{\textrm{rd}}/\tau _{\textrm{md}}=20$. The data for $\tau _{\textrm{rd}}/\tau _{\textrm{md}}=2.5$, 5, and 20 at $\kappa _1/\kappa _0 = 4$ correspond to the snapshots in Fig. 2a–c, respectively. (**c**) Propagation velocity $v_{\textrm{wave}}$ of the wave.

## Results

### Membrane modeling

A fluid membrane is represented by a triangulated surface as employed in our previous study^21–23^. The binding of two types of proteins is considered: curvature-inducing proteins and regulatory proteins (Fig. 1a). The bending energy of the membrane is represented by Canham–Helfrich model^24,25^ modified for two-component systems^26^ and is expressed as $F_{\textrm{cv}} = \int f_{\textrm{cv}}\ {\textrm{d}}S$ with

1 $$\begin{aligned} f_{\textrm{cv}} = \frac{\kappa _0}{2} (2H)^2(1-u) + \frac{\kappa _1}{2} (2H-C_0)^2u, \end{aligned}$$

where *u* is the concentration of the curvature-inducing proteins ($u=1$ at maximum coverage) and *H* is the mean curvature of the membrane. The first and second terms in Eq. (1) give the bending energies of the unbound and bound membranes, respectively. The unbound membrane has the bending rigidity $\kappa _0=20k_{\textrm{B}}T$ and zero spontaneous curvature, where $k_{\textrm{B}}T$ is the thermal energy. The bound membrane has the bending rigidity $\kappa _1$ and a finite spontaneous curvature $C_0$. This value of $\kappa _0$ is typical for lipid membranes^27^ and $\kappa _1>\kappa _0$ is required for curvature generation^28^. The membrane properties are independent of the concentration *v* of regulatory proteins.

We incorporated a modified FitzHugh–Nagumo model as RD equations for *u* and *v* on the membrane as follows:

2 $$\begin{aligned} \tau _{\textrm{rd}} \frac{\partial u}{\partial t}= & {} f(u,v) - G \frac{\partial f_{\textrm{cv}}}{\partial u} + D_u \nabla ^2 u, \end{aligned}$$

3 $$\begin{aligned} \tau _{\textrm{rd}} \frac{\partial v}{\partial t}= & {} g(u,v), \end{aligned}$$

where $\tau _{\textrm{rd}}$ is the RD time unit and *G* is the magnitude of mechanochemical coupling. That is, the curvature-inducing proteins binds more in the case the binding reduces the bending energy. Unless specified, $G=0.002$ is used. The diffusion coefficient $D_u = 0.1\sigma ^2$, and $\nabla ^2$ is the two-dimensional Laplace-Beltrami operator where $\sigma$ is the average edge length of the triangular mesh. The FitzHugh–Nagumo model^29,30^ is modified to maintain $0<u<1$ as follows: $f(u,v)= 0.002/u - 0.005/(1-u) + 0.1(u-0.2) - v$ and $g(u,v)=0.001( 0.1 u - v)$. The proteins of *u* and *v* are the activator and inhibitor, respectively, and an excitation occurs along the nullcline for *u* as shown in Fig. 1b. In the absence of the excitation, the proteins maintain the constant concentrations (the fixed point depicted in Fig. 1b). Once excited, the binding of the curvature-inducing proteins (*u*) induces the binding of the regulatory proteins (*v*) and subsequently, a high concentration of *v* leads to the unbinding of the curvature-inducing proteins (see the arrows in Fig. 1b).

To initiate a wave, the proteins are bound on a narrow region of the membrane at initial states, as indicated by the snapshot in the top row of Fig. 2b. The regulatory proteins are set at the left side of the curvature-inducing proteins to generate a wave in the right direction. The membrane motion is solved by molecular dynamics (MD) with the Langevin thermostat, that is, thermal conduction is assumed to be sufficiently fast, so that a constant temperature is maintained. The time $\tau _{\textrm{md}}$, in which a free particle diffuses the distance $\sigma$, is the MD time unit. A detailed description of the methods is provided in the “Methods” section and Supplementary Information.

### Waves in straight tubes

**Figure 5:**
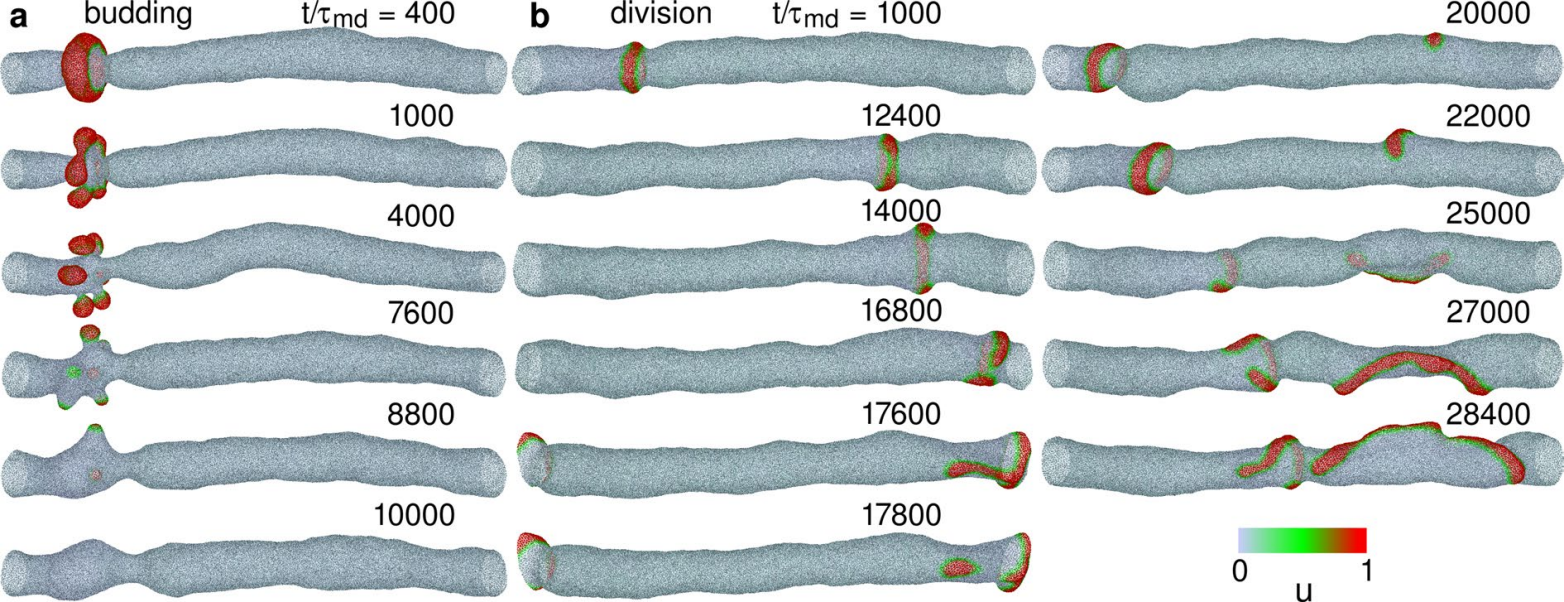
Sequential snapshots of RD waves in tubular membranes. (**a**) Formation of spherical buds for $G=0.002$, $\kappa _1/\kappa _0 = 2$, $C_0R_{\textrm{tube}}=6$, and $\tau _{\textrm{rd}}/\tau _{\textrm{md}}=20$. (**b**) Wave divides into two at $t/\tau _{\textrm{md}}=17800$, and subsequently, one of the waves rotates around the axis of the membrane tube for $G=0.004$, $\kappa _1/\kappa _0 = 1$, $C_0R_{\textrm{tube}}=5$, and $\tau _{\textrm{rd}}/\tau _{\textrm{md}}=2.5$.

**Figure 6:**
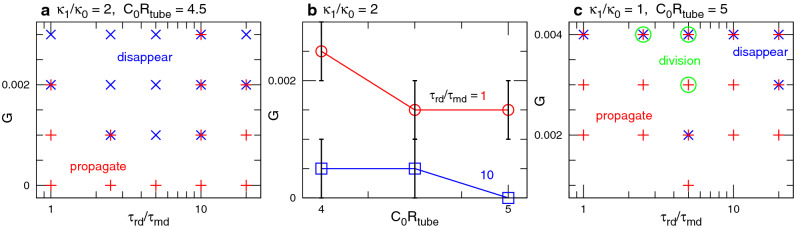
Dependence on the feedback strength *G*. (**a**) Phase diagram for $\kappa _1/\kappa _0 = 2$ and $C_0R_{\textrm{tube}}=4.5$. (**b**) Threshold value of *G* for wave disappearance at $\tau _{\textrm{rd}}/\tau _{\textrm{md}}=1$ and 10 for $\kappa _1/\kappa _0 = 2$. (**c**) Phase diagram for $\kappa _1/\kappa _0 = 1$ and $C_0R_{\textrm{tube}}=5$.

First, we describe the wave propagation in a straight membrane tube (Figs. 2, 3, 4, 5, 6). We investigate the conditions, in which the cylindrical shapes of unbound ($u=0.07$, unexcited) and bound ($u=0.9$, excited) membranes are stable and unstable, respectively; i.e., the region between two dashed lines in Fig. 3a. These two thresholds are determined by the condition that the homogeneous membrane generates a shrinking force along the tube axis given by^23,31^:

4 $$\begin{aligned} C_0R_{\textrm{tube}} < 1 + \frac{\kappa _0(1-u)}{\kappa _1 u}. \end{aligned}$$

Under the unstable condition (caused by high spontaneous curvature $C_0$ and/or more rigid proteins), the membrane exhibits buckling and buddings to a pearl-necklace shape. In our simulations, the tube radius $R_{\textrm{tube}}=9.79\sigma$.

Since the membrane ends are connected by a periodic boundary, a wave repeatedly propagates on the membrane, when the membrane deformation is small (see Figs. 2a and 4a and Supplementary Movie 1). However, the protein-binding induced deformation can generate a narrow neck in front of the wave and erases the wave (see Fig. 2b). The condition of this wave disappearance strongly depends on the time ratio $\tau _{\textrm{rd}}/\tau _{\textrm{md}}$ of RD to membrane deformation. For very fast wave propagation at $\tau _{\textrm{rd}}/\tau _{\textrm{md}}=1$, the membrane is only slightly deformed even at a high bending rigidity of the bound membrane ($\kappa _1/\kappa _0=5$) for $C_0R_{\textrm{tube}}=3$ (see Fig. 3b). Interestingly, a reentrant transition is observed; as $\tau _{\textrm{rd}}/\tau _{\textrm{md}}$ increases, waves disappear first and then propagate again (see Fig. 3b and c). A slow wave propagation allows the bound membrane to form a narrow tube with a roughly constant length because the membrane can relax into a thermal-equilibrium shape (see Figs. 2c and 4b and Supplementary Movie 2). A more rigid bound membrane forms a shorter tube (see Fig. 4b).

In undeformed tubes, the wave velocity $v_{\textrm{wave}}$ is proportional to $1/\tau _{\textrm{rd}}$. However, the membrane deformation slows down the waves so that $v_{\textrm{wave}}$ decreases with increasing $\kappa _1$ and $C_0$ (see Fig. 4c). Note that the wave propagation has hysteresis around the phase boundaries; that is, when the wave of a narrow tube, as shown in the bottom three snapshots in Fig. 2c, is used for an initial state, the wave propagates for a slightly faster RD of $\tau _{\textrm{rd}}/\tau _{\textrm{md}}=10$ in most cases (five of six runs), but the wave disappears for $\tau _{\textrm{rd}}/\tau _{\textrm{md}}=5$ at $\kappa _1/\kappa _0 = 4$ and $C_0R_{\textrm{tube}}=3$ (see Supplementary Movie 3).

At high $C_0$ and long $\tau _{\textrm{rd}}$, several spherical buds are formed before the disappearance (see Fig. 5a and Supplementary Movie 4). The excited membranes divide into several small domains and each domain forms a bud. Since the excited domains are separated from the membrane tube by narrow necks, the waves are stopped and membrane returns back into a cylindrical shape. Although the membrane topology is fixed in our simulation, vesicle formation from the buds are expected if topological changes are allowed as in Refs. ^32^ and^33^.

Next, we discuss the dependence on the feedback constant *G*. Since the nullcline for *u* shifts downwards with increasing *G*, waves become slower (see Supplementary Fig. 1) and eventually disappear at high *G* even for $\tau _{\textrm{rd}}/\tau _{\textrm{md}}=1$ (see Fig. 6). Interestingly, a wave division is observed at high $C_0$ (indicated by $\circ$ in Fig. 6c): a wave changes its shape from a ring to a strip and subsequently, a spot-shaped excited domain is pinched off; the spot grows to a long strip and rotates around the tube axis (see Fig. 5b and Supplementary Movie 5). Thus, wave propagation can change into an azimuthal direction through membrane deformation.

To examine the robustness of these dynamics, we simulate a tubular vesicle with the reduced volume $V^*= V/(4\pi {R_{\textrm{ves}}}^3/3) = 0.4$ where $R_{\textrm{ves}}= \sqrt{A/4\pi }=25.5\sigma$, and *V* and *A* are the volume and surface area of the vesicle, respectively. Similar wave dynamics are observed in a tubular vesicle as shown in Supplementary Figs. 2 and 3 and Supplementary Movies 6 and 7. A difference is that the vesicle length is finite so that the wave is terminated at the vesicle end. Since a free-standing vesicle can bend significantly in fluctuation, the cooccurrence region in the phase diagram is larger than the membrane tube (compare Supplementary Fig. 3 and Fig. 6). The wave rotation around the tube axis before the disappearance is also obtained at high *G* as in the membrane tube (see Supplementary Figs. 2b and Supplementary Movie 6).

**Figure 7:**
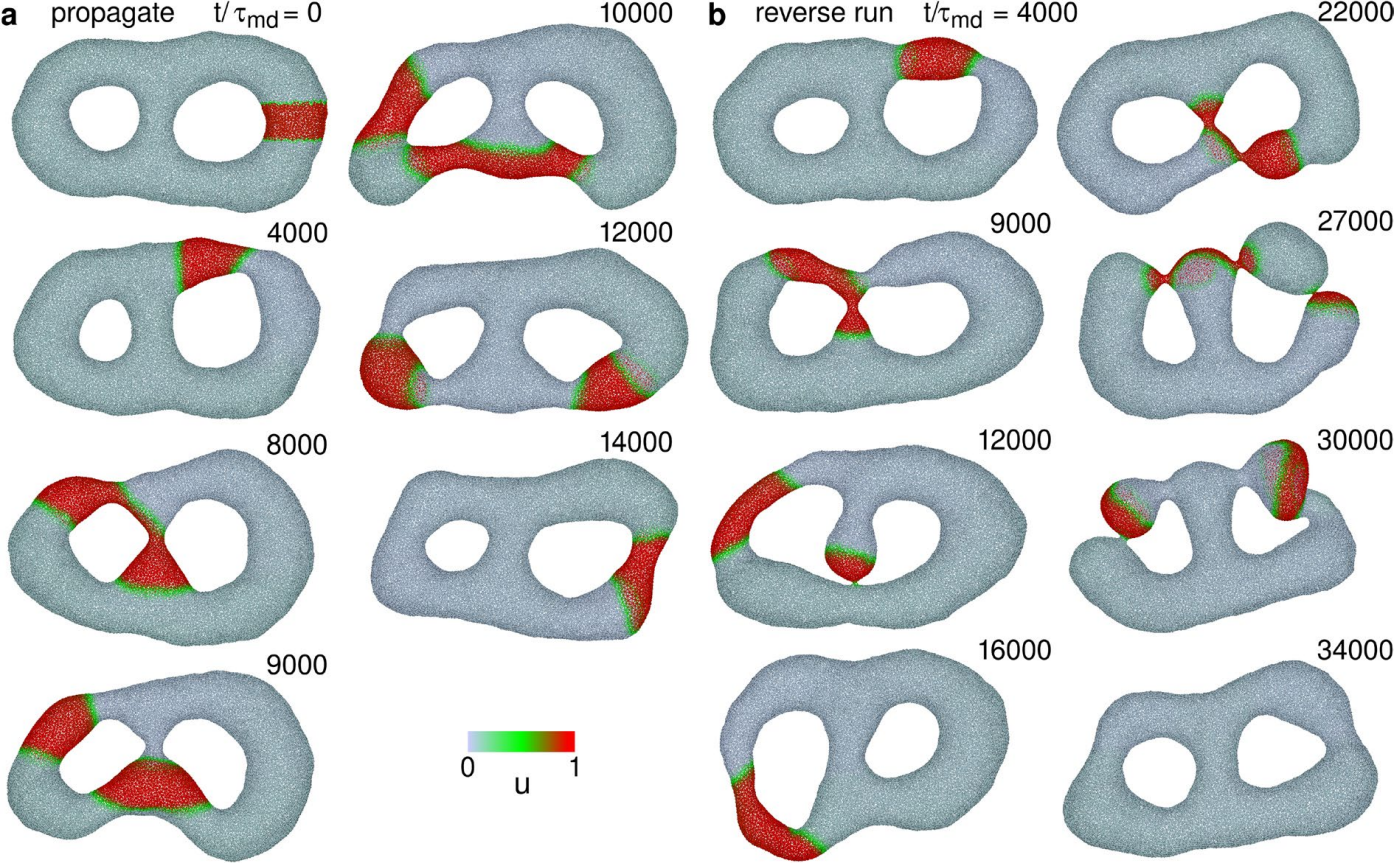
Sequential snapshots of RD waves in genus-2 vesicles for $G=0.002$, $\kappa _1/\kappa _0 = 4$, and $\tau _{\textrm{rd}}/\tau _{\textrm{md}}=5$. (**a**) Wave propagation for $C_0R_{\textrm{ves}}=6$. Waves go through both branches at the branching points. (**b**) Wave propagation for $C_0R_{\textrm{ves}}=8$. Waves occasionally disappear at narrow membrane necks and stochastically propagate in a reverse direction.

**Figure 8:**
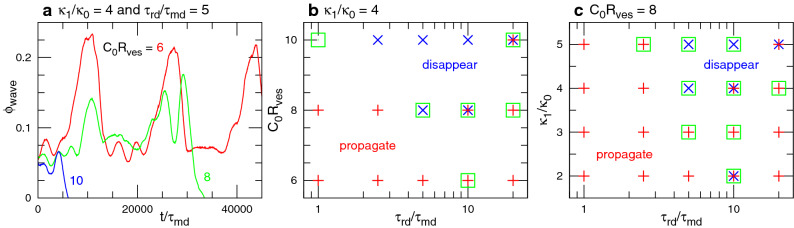
Wave propagation in genus-2 vesicles for $G=0.002$. (**a**) Time evolution of area fraction $\phi _{\textrm{wave}}$ of the excited region ($u>0.5$) for $\kappa _1/\kappa _0 = 4$ and $\tau _{\textrm{rd}}/\tau _{\textrm{md}}=5$. The data for $C_0R_{\textrm{ves}}=6$ and 8 correspond to those shown in Fig. 7a and b, respectively. (**b**) Phase diagram for $\kappa _1/\kappa _0 = 4$. (**c**) Phase diagram for $C_0R_{\textrm{ves}}=8$. The symbol $+$ indicates the wave propagation along both branches. The symbol $\times$ indicates the immediate wave disappearance before reaching the first branching point. The symbol $\Box$ indicates the partial wave propagation, in which one of the waves disappears at least before the wave reaches the original position.

### Waves in tubular networks

In living cells, organelles, such as ER and Golgi apparatus, have branched tubular networks^34–36^. They can be considered as a circuit for RD waves. Theoretically, multiple states can coexist in such networks^37,38^. As the simplest tubular network, we consider a genus-2 vesicle, which has two branching points, at $V^*= 0.4$, as shown in Fig. 7. Vesicles with two and higher genus can be produced experimentally^39–42^, and their shapes can be reproduced using the simulation method used in this study^42^.

For small-deformation conditions, a wave is divided into two at the branching points. Both the waves propagate separately (see Fig. 7a); subsequently, they collide in the left tube branch and disappear together (see the right snapshots in Fig. 7a); the remaining wave returns to the original position. These division and collision dynamics occur repeatedly (see Fig. 8a and Supplementary Movie 8). Similar to straight tubes, the waves in tubular networks disappear before reaching the first branching point at high $\kappa _1$ and/or high $C_0$ (see the line for $C_0R_{\textrm{ves}}=10$ in Fig. 8a and the phase diagram in Fig. 8b and c). In intermediate conditions, waves occasionally disappear; however, some of the waves stochastically continue to propagate (see Figs. 7b and  8). In this case, waves often propagate in the opposite direction: for example, Fig 7b shows that the wave propagates upwards in the middle branch and clockwise in the right branch. Thus, the wave pathways are stochastically changed by membrane deformation.

## Discussion

In this study, we have shown that the membrane deformation generated by the wave propagation and feedback to the wave exhibit characteristic behaviors, which are not seen in undeformable surfaces. When the wave propagates faster than the membrane deformation, the deformation is minimal. Conversely, for slower wave propagation, the membrane forms a thermal-equilibrium shape for the excited domain. When the shape of equilibrium is a narrow cylindrical tube locally, the excited domain moves translationally while retaining its shape. However, when spherical buds are formed, the wave disappears. In the case of the compatible speeds, the formation of membrane neck erases the wave. When the feedback of deformation to the protein binding is strong, the wave can be destabilized owing to the modification of reaction equation. In this case, a strip shape of waves can be formed, and subsequently, wave division and azimuthal rotation occur. Thus, reaction waves exhibit various dynamics due to the coupling of membrane deformation.

In this study, unbound membranes are considered as laterally homogeneous. However, biomembranes are heterogeneous and lipid rafts are formed as a platform of the functions^43^. Hence, different wave modes can be generated depending on the position of membrane in living cells. For example, the wave propagation induces membrane budding and subsequent vesicle formation at specific membrane locations. It was reported that endocytosis is enhanced in the presence of propagating waves of curvature-inducing proteins^44^. In living cells, vesicles are generated in ER, Golgi apparatus, and plasma membranes in intracellular membrane traffic^15,16,35^. In particular, ER exhibits necking, and its contacts with mitochondria may trigger chemical waves^35,36^. Thus, the wave-induced membrane deformation can play a role in ER and other organelles.

The network structures of membranes are observed *in vitro* and *in vivo*. They can work as the circuits for RD waves. We have demonstrated that membrane deformation can change the propagation pathways in genus-2 vesicles. This suggests that the deformation of membrane tubes or other soft substrates, such as gels, can be used as a tool to switch the wave circuits. Moreover, such switches may play a role in control signal transductions in living cells.

## Methods

The membrane tubes and vesicles are represented by triangular meshes of $N=15000$ and 10000 vertices, respectively. The membrane potentials are discretized as in Ref. ^45^ (see Supplementary Information). To produce a lateral fluidity, the meshes are stochastically reconstructed by a bond-flip Monte Carlo method^45,46^. Straight membrane tubes lie along the *x* axis with a length of $L_{x}=200\sigma$. An initial state is set up as follows: first, the membrane tube is equilibrated in the absence of excitation. Then, the protein concentration *u* is locally raised by 0.8 for $20<(x-x_{\textrm{end}})/\sigma <40$, and *v* by $0.00125(x-x_{\textrm{end}})/\sigma$ for $0<(x-x_{\textrm{end}})/\sigma \le 20$ and by $0.00125(40-x+x_{\textrm{end}})/\sigma$ for $20<(x-x_{\textrm{end}})/\sigma <40$, where $x_{\textrm{end}}$ is the left end position of the membrane tube. For tubular and genus-2 vesicles, initial states are similarly set up (see Supplementary Information).

We consider the membrane vertices of $u>0.5$ as the excited wave region. The center position $x_{\textrm{wave}}$ and width $w_{\textrm{wave}}$ of the wave are calculated by $x_{\textrm{wave}} = \sum _{u_{i}>0.5} x_i/N_{\textrm{wave}}$ and $w_{\textrm{wave}}^2 = \sum _{u_{i}>0.5} (x_i-x_{\textrm{wave}})^2/N_{\textrm{wave}}$, where $N_{\textrm{wave}}$ is the number of vertices belonging to the wave region. The area ratio of the wave is obtained as $\phi _{\textrm{wave}}=N_{\textrm{wave}}/N$. Error bars are calculated from three or more independent runs. To calculate the phase diagrams, six and ten simulation runs are conducted for regions close to the phase boundaries of wave disappearance and division, respectively. Additional details about the methods are described in Supplementary Information.

## Supplementary Information

Supplementary Information 1.

Supplementary Information 2.

Supplementary Information 3.

Supplementary Information 4.

Supplementary Information 5.

Supplementary Information 6.

Supplementary Information 7.

Supplementary Information 8.

Supplementary Information 9.

Supplementary Information 10.

## Data Availability

All data generated or analysed during this study are included in this published article [and its supplementary information files].

## References

[1] Yang Y, Wu M (2018). Rhythmicity and waves in the cortex of single cells. Philos. Trans. R. Soc. B.

[2] Gov NS (2018). Guided by curvature: Shaping cells by coupling curved membrane proteins and cytoskeletal forces. Philos. Trans. R. Soc. B.

[3] Chen C-H, Tsai F-C, Wang C-C, Lee C-H (2009). Three-dimensional characterization of active membrane waves on living cells. Phys. Rev. Lett..

[4] Banerjee T (2022). Spatiotemporal dynamics of membrane surface charge regulates cell polarity and migration. Nat. Cell Biol..

[5] Taniguchi D (2013). Phase geometries of two-dimensional excitable waves govern self-organized morphodynamics of amoeboid cells. Proc. Natl. Acad. Sci. USA.

[6] Kondo S, Miura T (2010). Reaction-diffusion model as a framework for understanding biological pattern formation. Science.

[7] Manukyan L, Montandon SA, Fofonjka A, Smirnov S, Milinkovitch MC (2017). A living mesoscopic cellular automaton made of skin scales. Nature.

[8] Krause AL, Gaffney EA, Maini PK, Klika V (2021). Modern perspectives on near-equilibrium analysis of Turing systems. Philos. Trans. R. Soc. A.

[9] Frank JR, Guven J, Kardar M, Shackleton H (2019). Pinning of diffusional patterns by non-uniform curvature. EPL.

[10] Nishide R, Ishihara S (2022). Pattern propagation driven by surface curvature. Phys. Rev. Lett..

[11] Sánchez-Garduño F, Krause AL, Castillo JA, Padilla P (2019). Turing-Hopf patterns on growing domains: The torus and the sphere. J. Theor. Biol..

[12] McMahon HT, Gallop JL (2005). Membrane curvature and mechanisms of dynamic cell membrane remodelling. Nature.

[13] Baumgart T, Capraro BR, Zhu C, Das SL (2011). Thermodynamics and mechanics of membrane curvature generation and sensing by proteins and lipids. Annu. Rev. Phys. Chem..

[14] Suetsugu S, Kurisu S, Takenawa T (2014). Dynamic shaping of cellular membranes by phospholipids and membrane-deforming proteins. Physiol. Rev..

[15] McMahon HT, Boucrot E (2011). Molecular mechanism and physiological functions of clathrin-mediated endocytosis. Nat. Rev. Mol. Cell. Biol..

[16] Johannes L, Parton RG, Bassereau P, Mayor S (2015). Building endocytic pits without clathrin. Nat. Rev. Mol. Cell. Biol..

[17] Wu Z, Su M, Tong C, Wu M, Liu J (2018). Membrane shape-mediated wave propagation of cortical protein dynamics. Nat. Commun..

[18] Litschel T, Ramm B, Maas R, Heymann M, Schwille P (2018). Beating vesicles: Encapsulated protein oscillations cause dynamic membrane deformations. Angew. Chem..

[19] Christ S, Litschel T, Schwille P, Lipowsky R (2021). Active shape oscillations of giant vesicles with cyclic closure and opening of membrane necks. Soft Matter.

[20] Peleg B, Disanza A, Scita G, Gov N (2011). Propagating cell-membrane waves driven by curved activators of actin polymerization. PLoS One.

[21] Tamemoto N, Noguchi H (2020). Pattern formation in reaction-diffusion system on membrane with mechanochemical feedback. Sci. Rep..

[22] Tamemoto N, Noguchi H (2021). Reaction-diffusion waves coupled with membrane curvature. Soft Matter.

[23] Tamemoto N, Noguchi H (2022). Excitable reaction-diffusion waves of curvature-inducing proteins on deformable membrane tubes. Phys. Rev. E.

[24] Canham PB (1970). The minimum energy of bending as a possible explanation of the biconcave shape of the human red blood cell. J. Theor. Biol..

[25] Helfrich W (1973). Elastic properties of lipid bilayers: Theory and possible experiments. Z. Naturforsch.

[26] Noguchi H (2021). Vesicle budding induced by binding of curvature-inducing proteins. Phys. Rev. E.

[27] Dimova R (2014). Recent developments in the field of bending rigidity measurements on membranes. Adv. Colloid Interface Sci..

[28] Noguchi H (2022). Binding of curvature-inducing proteins onto biomembranes. Int. J. Mod. Phys. B.

[29] FitzHugh R (1961). Impulses and physiological states in theoretical models of nerve membrane. Biophys. J..

[30] Nagumo J, Arimoto S, Yoshizawa S (1962). An active pulse transmission line simulating nerve axon. Proc. IRE.

[31] Ou-Yang ZC, Helfrich W (1989). Bending energy of vesicle membranes: General expressions for the first, second, and third variation of the shape energy and applications to spheres and cylinders. Phys. Rev. A.

[32] Gompper G, Kroll DM (1998). Membranes with fluctuating topology: Monte Carlo simulations Phys. Rev. Lett..

[33] Tachikawa M, Mochizuki A (2017). Golgi apparatus self-organizes into the characteristic shape via postmitotic reassembly dynamics. Proc. Natl. Acad. Sci. USA.

[34] Heinrich L (2021). Whole-cell organelle segmentation in volume electron microscopy. Nature.

[35] Zucker B, Kozlov MM (2022). Mechanism of shaping membrane nanostructures of endoplasmic reticulum. Proc. Natl. Acad. Sci. USA.

[36] Zhao Y (2022). Isotropic super-resolution light-sheet microscopy of dynamic intracellular structures at subsecond timescales. Nat. Methods.

[37] Nakao H, Mikhailov AS (2010). Turing patterns in network-organized activator-inhibitor systems. Nat. Phys..

[38] Vespignani A (2012). Modelling dynamical processes in complex socio-technical systems. Nat. Phys..

[39] Michalet X, Bensimon D (1995). Observation of stable shapes and conformal diffusion in genus 2 vesicles. Science.

[40] Haluska CK, Góźdź WT, Döbereiner H-G, Förster S, Gompper G (2002). Giant hexagonal superstructures in diblock-copolymer membranes. Phys. Rev. Lett..

[41] Akashi K-I, Miyata H (2010). Lipid bilayer vesicles with numbers of membrane-linking pores. J. Phys. Soc. Jpn..

[42] Noguchi H, Sakashita A, Imai M (2015). Shape transformations of toroidal vesicles. Soft Matter.

[43] Lingwood D, Simons K (2010). Lipid Rafts as a membrane-organizing principle. Science.

[44] Yang Y, Xiong D, Pipathsouk A, Weiner OD, Wu M (2017). Clathrin assembly defines the onset and geometry of cortical patterning. Dev. Cell.

[45] Noguchi H, Gompper G (2005). Dynamics of fluid vesicles in shear flow: Effect of membrane viscosity and thermal fluctuations. Phys. Rev. E.

[46] Gompper G, Kroll DM (1997). Network models of fluid, hexatic and polymerized membranes. J. Phys. Condens. Matter.

