# Supplementary material for: Disappearance, division, and route change of excitable reaction-diffusion waves in deformable membranes
Source: Sci Rep. 2023 Apr 17;13:6207. doi: 10.1038/s41598-023-33376-9 (PMC10110617; doi:10.1038/s41598-023-33376-9)
Supplement: Supplementary file 1 — Supplementary Information 1. [file 41598_2023_33376_MOESM1_ESM.pdf]

Supplementary Information for
Disappearance, division, and route change of excitable reaction-diffusion waves in deformable membranes

Hiroshi Noguchi
Institute for Solid State Physics, University of Tokyo,
Kashiwa, Chiba 277-8581, Japan
Correspondence to: noguchi@issp.u-tokyo.ac.jp

METHOD DETAILS

Membrane motion is solved by MD using a Langevin thermostat:

$$m\frac{\partial^2 \boldsymbol{r}_i}{\partial t^2} = -\frac{\partial U}{\partial \boldsymbol{r}_i} - \zeta\frac{\partial \boldsymbol{r}_i}{\partial t} + \boldsymbol{g}_i(t), \quad \text{(S1)}$$

where m is the mass of the membrane vertex. The friction coefficient ζ and Gaussian white noise $\boldsymbol{g}_i$ obey the fluctuation-dissipation theorem. The MD time unit is given by $\tau_{\rm md} = \zeta\sigma^2/k_{\rm B}T$. We used the potential $U = U_{\rm ar} + U_{\rm bond} + U_{\rm rep} + U_{\rm cv}$ for a tubular membrane, where $U_{\rm ar}$ is the constraint potential for surface area A; $U_{\rm bond}$ and $U_{\rm rep}$ are the bond and repulsive potentials, respectively; $U_{\rm cv}$ is the discretized potential for the bending energy, $F_{\rm cv}$, using the dual lattice.

$$U_{\rm ar} = \frac{k_{\rm ar}}{2}(A - A_0)^2, \quad \text{(S2)}$$

$$U_{\rm bond} = \sum_{\rm bond} \frac{b\exp\left(\frac{1}{l_{\rm c0}-r_{i,j}}\right)}{l_{\rm max} - r_{i,j}}\Theta(r_{i,j} - l_{\rm c0}), \quad \text{(S3)}$$

$$U_{\rm rep} = \sum_{\rm all\ pairs} \frac{b\exp\left(\frac{1}{r_{i,j}-l_{\rm c1}}\right)}{r_{i,j} - l_{\rm min}}\Theta(l_{\rm c1} - r_{i,j}), \quad \text{(S4)}$$

$$U_{\rm cv} = \frac{\kappa_0}{2}(1-u)\sum_i \frac{1}{s_i}\left(\sum_{j(i)} \frac{s_{i,j}\mathbf{r}_{i,j}}{r_{i,j}}\right)^2 \quad \text{(S5)}$$
$$+\frac{\kappa_1}{2}u\sum_i s_i\left(\frac{1}{s_i}\sum_{j(i)} \frac{s_{i,j}\mathbf{r}_{i,j}}{r_{i,j}} - C_0\mathbf{n}_i\right)^2,$$

where $\Theta(x)$ is the unit step function, $r_{i,j}$ is the distance between two vertices i and j, and $\mathbf{n}_i$ is the normal vector at the i-th vertex. The sum over $j(i)$ is over the neighbors of the i-th vertex, which are connected by bonds. The length of a bond in the dual lattice is $s_{i,j} = r_{i,j}[\cot(\theta_1) + \cot(\theta_2)]/2$, where the angles θ_1 and θ_2 are opposite to bond ij in the two triangles sharing this bond. $s_i = \sum_{j(i)} s_{i,j}r_{i,j}/4$ is the area of the dual cell of vertex i. In this study, we used $k_{\rm ar} = 4k_{\rm B}T$, $b = 80k_{\rm B}T$, $l_{\rm max} = 1.33\sigma$, $l_{\rm c0} = 1.15\sigma$, $l_{\rm c1} = 0.85\sigma$, and $l_{\rm min} = 0.67\sigma$. The details of membrane potentials are provided in Ref. 1.

For tubular and genus-2 vesicles, a volume constraint potential $U_{\rm vol}$ is added to maintain the reduced volume V^* of the vesicle:

$$U_{\rm vol} = \frac{k_{\rm vol}}{2}(V - V_0)^2. \quad \text{(S6)}$$

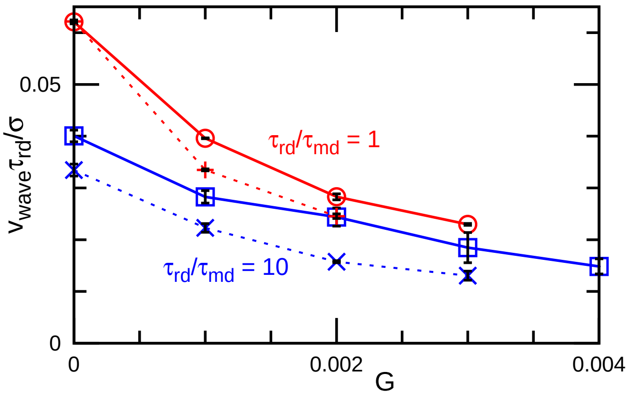

Supplementary Fig. 1. Velocity $v_{\rm wave}$ of the wave propagation in tubular membranes at $\tau_{\rm rd}/\tau_{\rm md} = 1$ ($\circ, +$) and $\tau_{\rm rd}/\tau_{\rm md} = 10$ ($\square, \times$) for $G = 0.002$ and $\kappa_1/\kappa_0 = 2$. The solid and dashed lines represent the data for $C_0R_{\rm tube} = 4$ and 4.5, respectively.

In this study, we used $k_{\rm vol} = 2k_{\rm B}T$ and set the reduced volume $V^* = 0.4$ both for tubular and genus-2 vesicles.

Equations (2) and (3) are numerically integrated using the forward difference method for time and finite-volume scheme for space[2] with a time step of $\Delta t_{\rm rd} = 0.0001\tau_{\rm md}$. Equation (S1) is integrated using the leapfrog method with a time step of $\Delta t_{\rm md} = 0.001\tau_{\rm md}$. In the bond-flip process[1,3], a bond is flipped into the bond connecting the diagonal vertices of two neighboring triangles by the Metropolis Monte Carlo method. This flip process is performed for one per membrane bond with a time step of $0.02\tau_{\rm md}$.

Initial states of vesicles are set up as follows: first, the vesicles are equilibrated in the absence of excitation. Then, the gyration tensor of the vesicles $a_{\alpha\beta} = (1/N)\sum_i(\alpha_i - \alpha_{\rm G})(\beta_i - \beta_{\rm G})$ are calculated, where $\alpha_{\rm G}$ is the α component of the center of mass and $\alpha, \beta \in \{x, y, z\}$. Next, the centers of the vesicles are set as the origin of coordinates $[(x_{\rm G}, y_{\rm G}, z_{\rm G}) = (0, 0, 0)]$, and the vesicles are rotated to orient the eigenvectors for the maximum, middle, and minimum eigenvalues along the x, y, z axes, respectively. For the tubular vesicle, protein concentration u is locally raised by 0.8 for the left vesicle end ($x < -70\sigma$); however, v is not modified (see the top snapshots in Fig. 2). For the genus-2 vesicle, u is locally raised by 0.8 for the right middle region ($x > 15\sigma$ and $-5\sigma < y < 5\sigma$), and v by $0.005(y + 10)/\sigma$ for $-10\sigma < y \leq -5\sigma$ and by $0.0025(5 - y)/\sigma$ for

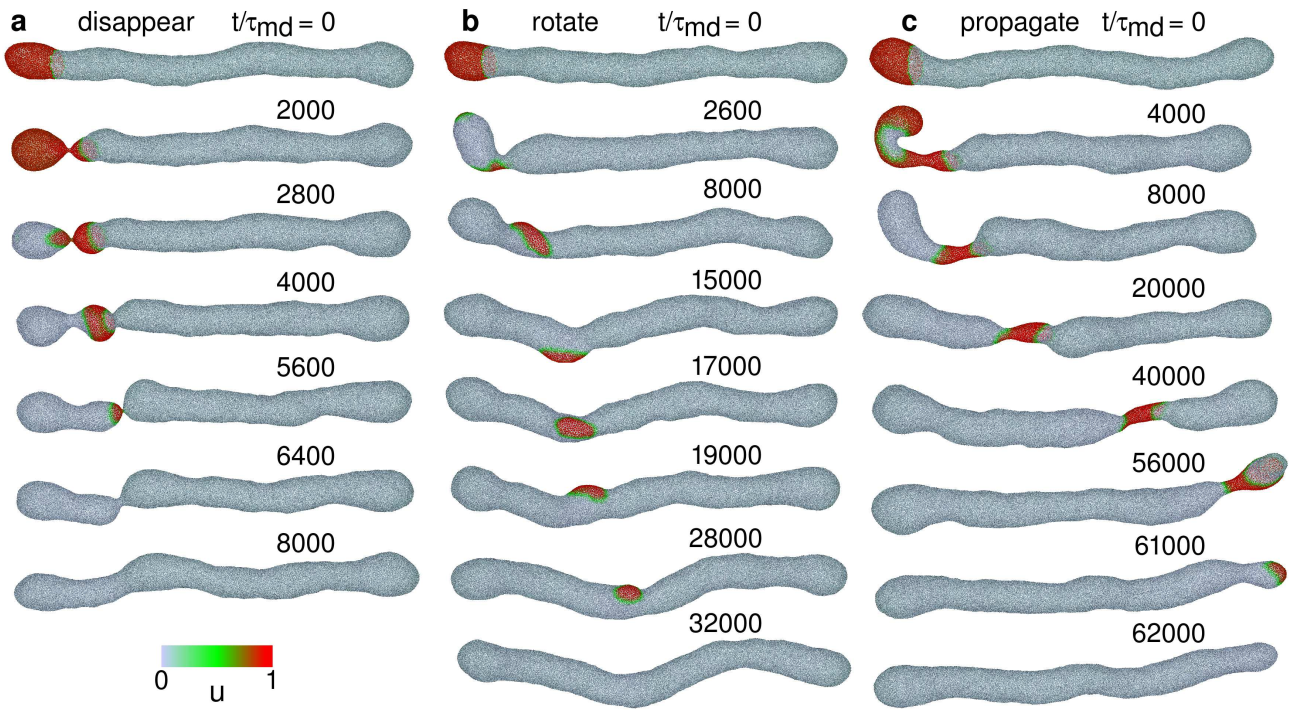

Supplementary Fig. 2. Sequential snapshots of RD wave in tubular vesicles at $\kappa_1/\kappa_0 = 4$ and $C_0R_{\rm ves} = 8$. **a** Wave disappears at $t/\tau_{\rm md} = 6200$ for $G = 0.004$ and $\tau_{\rm rd}/\tau_{\rm md} = 5$. **b** Wave rotates around the tubular axis and eventually disappears at $t/\tau_{\rm md} = 30800$ for $G = 0.005$ and $\tau_{\rm rd}/\tau_{\rm md} = 5$. **c** Wave propagates, thus forming a narrow tube of the bound membrane for $G = 0.005$ and $\tau_{\rm rd}/\tau_{\rm md} = 20$. The concentration u of curvature-inducing proteins is indicated in different colors (see the color bar in the bottom left).

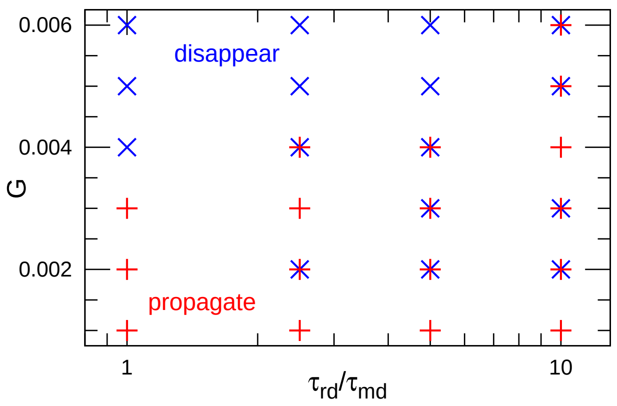

Supplementary Fig. 3. Phase diagram of tubular vesicles at $\kappa_1/\kappa_0 = 4$ and $C_0R_{\rm ves} = 8$. The symbols $+$ and $\times$ represent wave propagation and disappearance, respectively. Both the symbols are overlayed, when both dynamics are observed in six simulation runs.

$-5\sigma < y < 5\sigma$ at $x > 15\sigma$ (see the top snapshot in Fig. 7a).

MOVIE CAPTIONS

Supplementary Movie 1. Wave propagation in a membrane tube for $\tau_{\rm rd}/\tau_{\rm md} = 2.5$ at $G = 0.002$, $\kappa_1/\kappa_0 = 4$, and $C_0R_{\rm tube} = 3$. Corresponding to Fig. 3a.

Supplementary Movie 2. Wave propagation in a membrane tube for $\tau_{\rm rd}/\tau_{\rm md} = 20$ at $G = 0.002$, $\kappa_1/\kappa_0 = 4$, and $C_0R_{\rm tube} = 3$. Corresponding to Fig. 3c.

Supplementary Movie 3. Wave disappearance in a membrane tube for $\tau_{\rm rd}/\tau_{\rm md} = 5$ at $G = 0.002$, $\kappa_1/\kappa_0 = 4$, and $C_0R_{\rm tube} = 3$. A locally narrow-tube shape formed at $\tau_{\rm rd}/\tau_{\rm md} = 20$ is used as an initial state.

Supplementary Movie 4. Formation of spherical buds for $G = 0.002$, $\kappa_1/\kappa_0 = 2$, $C_0R_{\rm tube} = 6$, and $\tau_{\rm rd}/\tau_{\rm md} = 20$. Corresponding to Fig. 5a.

Supplementary Movie 5. Wave division and rotation of strip-shaped wave in a membrane tube for $G = 0.004$, $\kappa_1/\kappa_0 = 1$, $C_0R_{\rm tube} = 5$, and $\tau_{\rm rd}/\tau_{\rm md} = 2.5$. Corresponding to Fig. 5b.

Supplementary Movie 6. Disappearance of a rotating spot-shaped wave in a tubular vesicle for $\tau_{\rm rd}/\tau_{\rm md} = 5$ at $G = 0.005$, $\kappa_1/\kappa_0 = 4$, and $C_0R_{\rm ves} = 8$. Corresponding

to Supplementary Fig. 2b.

Supplementary Movie 7. Wave propagation in a tubular vesicle for $\tau_{\rm rd}/\tau_{\rm md} = 20$ at $G = 0.005$, $\kappa_1/\kappa_0 = 4$, and $C_0R_{\rm ves} = 8$. Corresponding to Supplementary Fig. 2c.

Supplementary Movie 8. Wave propagation in a genus-2 vesicle for $C_0R_{\rm ves} = 6$ at $G = 0.002$, $\kappa_1/\kappa_0 = 4$, and $\tau_{\rm rd}/\tau_{\rm md} = 5$. Corresponding to Fig. 7a.

Supplementary Movie 9. Wave propagation in a genus-2 vesicle for $C_0R_{\rm ves} = 8$ at $G = 0.002$, $\kappa_1/\kappa_0 = 4$, and $\tau_{\rm rd}/\tau_{\rm md} = 5$. Corresponding to Fig. 7b.

[1] Noguchi, H. & Gompper, G. Dynamics of fluid vesicles in shear flow: Effect of membrane viscosity and thermal fluctuations. *Phys. Rev. E* **72**, 011901 (2005).

[2] Tamemoto, N. & Noguchi, H. Pattern formation in reaction-diffusion system on membrane with mechanochemical feedback. *Sci. Rep.* **10**, 19582 (2020).

[3] Gompper G. & Kroll, D. M. Network Models of Fluid, Hexatic and Polymerized Membranes. *J. Phys. Condens. Matter* **9**, 8795–8834 (1997).
